# (*Z*)-4-[(2-Amino­anilino)(phen­yl)methyl­idene]-3-methyl-1-phenyl-1*H*-pyrazol-5(4*H*)-one

**DOI:** 10.1107/S1600536811037470

**Published:** 2011-09-30

**Authors:** Rong Lu, Hua Xia, Xingqiang Lü, Shunsheng Zhao

**Affiliations:** a College of Chemical Engineering, Northwest University, Xi’an 710069, Shaanxi, People’s Republic of China; b College of Chemistry and Chemical Engineering, Xian University of Science and Technology, Xi’an 710054, Shaanxi, People’s Republic of China

## Abstract

The mol­ecule of the title compound, C_23_H_20_N_4_O, assumes a non-planar conformation in which the pyrazolone ring forms dihedral angles of 10.33 (11), 65.34 (11) and 63.52 (10)° with the three benzene rings. In the crystal, the mol­ecules are linked by inter­molecular N—H⋯N hydrogen bonds, generating chains parallel to the *b* axis. The secondary amino group is involved in an intra­molecular N—H⋯O hydrogen bond.

## Related literature

For the synthesis, properties and applications of the title compound, see: Hennig & Mann (1988 ▶); Bao *et al.* (2005 ▶).
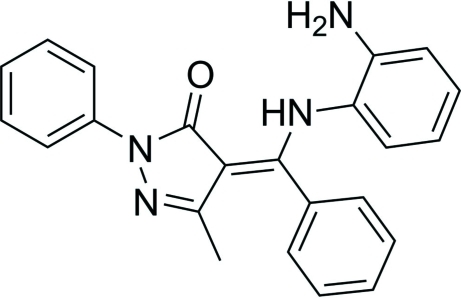


## Experimental

### 

#### Crystal data

C_23_H_20_N_4_O
*M* *_r_* = 368.43
Monoclinic, 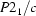

*a* = 9.200 (2) Å
*b* = 21.680 (5) Å
*c* = 9.608 (2) Å
β = 97.840 (4)°
*V* = 1898.4 (7) Å^3^
*Z* = 4
Mo *K*α radiation
μ = 0.08 mm^−1^
*T* = 273 K
0.30 × 0.20 × 0.20 mm

#### Data collection

Bruker SMART 1K CCD area-detector diffractometer
Absorption correction: multi-scan (*SADABS*; Sheldrick, 2004 ▶) *T*_min_ = 0.857, *T*_max_ = 1.000
9447 measured reflections
3369 independent reflections
1983 reflections with *I* > 2σ(*I*)
*R*_int_ = 0.039

#### Refinement

*R*[*F* ^2^ > 2σ(*F* ^2^)] = 0.043
*wR*(*F* ^2^) = 0.119
*S* = 0.94
3369 reflections
258 parameters
H atoms treated by a mixture of independent and constrained refinement
Δρ_max_ = 0.20 e Å^−3^
Δρ_min_ = −0.24 e Å^−3^

### 

Data collection: *SMART* (Bruker, 2001 ▶); cell refinement: *SAINT* (Bruker, 2001 ▶); data reduction: *SAINT*; program(s) used to solve structure: *SHELXS97* (Sheldrick, 2008 ▶); program(s) used to refine structure: *SHELXL97* (Sheldrick, 2008 ▶); molecular graphics: *SHELXTL* (Sheldrick, 2008 ▶); software used to prepare material for publication: *SHELXTL* and local programs.

## Supplementary Material

Crystal structure: contains datablock(s) I, global. DOI: 10.1107/S1600536811037470/fy2020sup1.cif

Structure factors: contains datablock(s) I. DOI: 10.1107/S1600536811037470/fy2020Isup2.hkl

Supplementary material file. DOI: 10.1107/S1600536811037470/fy2020Isup3.cml

Additional supplementary materials:  crystallographic information; 3D view; checkCIF report

## Figures and Tables

**Table 1:** Hydrogen-bond geometry (Å, °)

*D*—H⋯*A*	*D*—H	H⋯*A*	*D*⋯*A*	*D*—H⋯*A*
N3—H1*C*⋯O1	0.96 (2)	1.93 (2)	2.733 (2)	139.8 (16)
N4—H4*B*⋯N2^i^	0.86	2.34	3.194 (2)	173
N4—H4*A*⋯N2^ii^	0.86	2.50	3.209 (2)	140

Symmetry codes: (i) 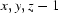; (ii) 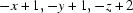.

**Fig. 1.**
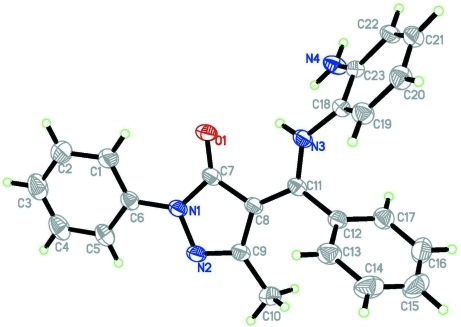
Molecular structure of (I), with atom labels and 30% probability displacement ellipsoids for non-H atoms.

**Fig. 2.**
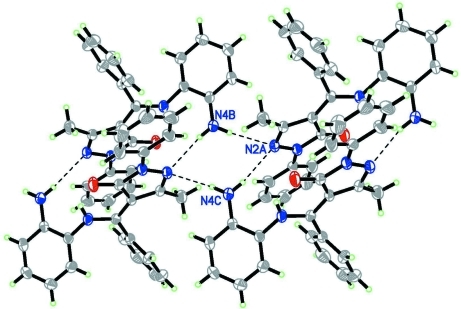
Packing of (I), showing molecules connected by N—H···N hydrogen bonds (dashed lines). H atoms not involved in hydrogen bonding have been omitted.

**Fig. 3.**
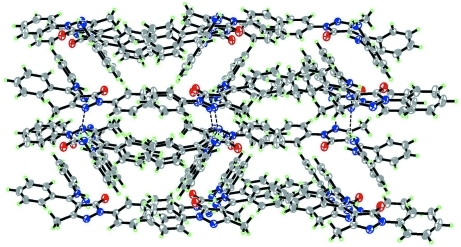
Packing of (I), showing assembly of molecules connected by stacking interaction.

## supplementary crystallographic information

### Comment

The molecules of the title compound (Fig. 1) are linked by N—H···N hydrogen bonds, generating parallel chains as shown in Fig. 2. The aminophenyl rings protrude on both sides of these chains. Adjacent chains are linked by stacking interactions between the protruding rings. The distance between the ring centroids is 3.6953 (14) Å.

### Experimental

The title compound was obtained according to the synthetic procedure of Hennig & Mann (1988). *o*-Phenylenediamine and 4-benzoyl-3-methyl-1-phenyl-1*H*-pyrazol-5(4*H*)-one were refluxed for 2 h in a 1:1 ratio in absolute ethanol to give the product. The single crystal suitble for X-ray diffraction was obtained by slow evaporation of the ethanolic solution of the title compound.

### Refinement

The H atom bonded to N3 was located in a difference map and refined freely. Other H atoms were positioned geometrically and refined using a riding model with N—H = 0.86 Å, C—H = 0.95–0.99 Å and with *U*_iso_(H) = 1.2 (1.5 for methyl groups) times *U*_eq_(C/N).

### Crystal data

**Table:**

C_23_H_20_N_4_O	*F*(000) = 776
*M**_r_* = 368.43	*D*_x_ = 1.289 Mg m^−^^3^
Monoclinic, *P*2_1_/*c*	Mo *K*α radiation, λ = 0.71073 Å
Hall symbol: -P 2ybc	Cell parameters from 10250 reflections
*a* = 9.200 (2) Å	θ = 1.9–25.1°
*b* = 21.680 (5) Å	µ = 0.08 mm^−^^1^
*c* = 9.608 (2) Å	*T* = 273 K
β = 97.840 (4)°	Block, orange
*V* = 1898.4 (7) Å^3^	0.30 × 0.20 × 0.20 mm
*Z* = 4	

### Data collection

**Table:**

Bruker SMART 1K CCD area-detector diffractometer	3369 independent reflections
Radiation source: fine-focus sealed tube	1983 reflections with *I* > 2σ(*I*)
graphite	*R*_int_ = 0.039
thin–slice ω scans	θ_max_ = 25.1°, θ_min_ = 1.9°
Absorption correction: multi-scan (*SADABS*; Sheldrick, 2004)	*h* = −8→10
*T*_min_ = 0.857, *T*_max_ = 1.000	*k* = −25→25
9447 measured reflections	*l* = −11→9

### Refinement

**Table:**

Refinement on *F*^2^	Primary atom site location: structure-invariant direct methods
Least-squares matrix: full	Secondary atom site location: difference Fourier map
*R*[*F*^2^ > 2σ(*F*^2^)] = 0.043	Hydrogen site location: inferred from neighbouring sites
*wR*(*F*^2^) = 0.119	H atoms treated by a mixture of independent and constrained refinement
*S* = 0.94	*w* = 1/[σ^2^(*F*_o_^2^) + (0.0677*P*)^2^] where *P* = (*F*_o_^2^ + 2*F*_c_^2^)/3
3369 reflections	(Δ/σ)_max_ < 0.001
258 parameters	Δρ_max_ = 0.20 e Å^−^^3^
0 restraints	Δρ_min_ = −0.24 e Å^−^^3^

### Special details

**Table:**

Geometry. All e.s.d.'s (except the e.s.d. in the dihedral angle between two l.s. planes) are estimated using the full covariance matrix. The cell e.s.d.'s are taken into account individually in the estimation of e.s.d.'s in distances, angles and torsion angles; correlations between e.s.d.'s in cell parameters are only used when they are defined by crystal symmetry. An approximate (isotropic) treatment of cell e.s.d.'s is used for estimating e.s.d.'s involving l.s. planes.
Refinement. Refinement of *F*^2^ against ALL reflections. The weighted *R*-factor *wR* and goodness of fit *S* are based on *F*^2^, conventional *R*-factors *R* are based on *F*, with *F* set to zero for negative *F*^2^. The threshold expression of *F*^2^ > σ(*F*^2^) is used only for calculating *R*-factors(gt) *etc*. and is not relevant to the choice of reflections for refinement. *R*-factors based on *F*^2^ are statistically about twice as large as those based on *F*, and *R*- factors based on ALL data will be even larger.

### Fractional atomic coordinates and isotropic or equivalent isotropic displacement parameters (Å^2^)

**Table:**

	*x*	*y*	*z*	*U*_iso_*/*U*_eq_	
N1	0.34922 (16)	0.43731 (6)	1.14992 (14)	0.0474 (4)	
O1	0.24396 (16)	0.42406 (6)	0.91531 (13)	0.0633 (4)	
N2	0.40425 (17)	0.48648 (7)	1.23643 (14)	0.0496 (4)	
N3	0.20658 (17)	0.53669 (7)	0.78768 (14)	0.0507 (5)	
N4	0.30500 (17)	0.51166 (7)	0.53720 (16)	0.0606 (5)	
H4A	0.3527	0.4993	0.6155	0.073*	
H4B	0.3353	0.5021	0.4592	0.073*	
C7	0.2924 (2)	0.45725 (8)	1.01684 (18)	0.0474 (5)	
C8	0.30147 (19)	0.52375 (8)	1.02509 (17)	0.0439 (5)	
C11	0.24301 (19)	0.56189 (8)	0.91417 (18)	0.0447 (5)	
C6	0.3536 (2)	0.37681 (8)	1.20613 (19)	0.0484 (5)	
C18	0.1294 (2)	0.56260 (8)	0.66247 (18)	0.0444 (5)	
C23	0.1803 (2)	0.54668 (8)	0.53687 (18)	0.0437 (5)	
C9	0.3752 (2)	0.53710 (8)	1.16249 (18)	0.0464 (5)	
C12	0.2170 (2)	0.62851 (8)	0.93519 (19)	0.0463 (5)	
C19	0.0030 (2)	0.59683 (9)	0.6613 (2)	0.0556 (5)	
H19A	−0.0302	0.6068	0.7457	0.067*	
C22	0.0969 (2)	0.56593 (8)	0.41134 (19)	0.0521 (5)	
H22A	0.1273	0.5553	0.3261	0.063*	
C20	−0.0747 (2)	0.61646 (9)	0.5361 (2)	0.0589 (6)	
H20A	−0.1581	0.6407	0.5360	0.071*	
C21	−0.0276 (2)	0.59984 (9)	0.4113 (2)	0.0560 (6)	
H21A	−0.0812	0.6118	0.3265	0.067*	
C10	0.4277 (2)	0.59724 (9)	1.2264 (2)	0.0634 (6)	
H10A	0.4739	0.5905	1.3209	0.095*	
H10B	0.3458	0.6247	1.2271	0.095*	
H10C	0.4971	0.6152	1.1722	0.095*	
C5	0.4291 (2)	0.36538 (10)	1.3386 (2)	0.0614 (6)	
H5A	0.4777	0.3973	1.3905	0.074*	
C1	0.2792 (3)	0.32924 (9)	1.1319 (2)	0.0670 (6)	
H1A	0.2265	0.3365	1.0438	0.080*	
C17	0.2816 (2)	0.67312 (9)	0.8609 (2)	0.0592 (6)	
H17A	0.3417	0.6617	0.7949	0.071*	
C14	0.0998 (3)	0.70835 (12)	1.0518 (3)	0.0808 (8)	
H14A	0.0365	0.7202	1.1147	0.097*	
C4	0.4318 (3)	0.30662 (12)	1.3930 (2)	0.0780 (7)	
H4C	0.4834	0.2991	1.4815	0.094*	
C16	0.2562 (3)	0.73473 (10)	0.8853 (3)	0.0779 (8)	
H16A	0.3003	0.7646	0.8357	0.094*	
C15	0.1671 (3)	0.75248 (12)	0.9814 (3)	0.0877 (9)	
H15A	0.1526	0.7941	0.9985	0.105*	
C2	0.2838 (3)	0.27058 (10)	1.1899 (3)	0.0884 (8)	
H2B	0.2341	0.2385	1.1396	0.106*	
C13	0.1247 (2)	0.64666 (10)	1.0308 (2)	0.0636 (6)	
H13A	0.0797	0.6171	1.0806	0.076*	
C3	0.3600 (3)	0.25895 (11)	1.3197 (3)	0.0867 (8)	
H3B	0.3630	0.2194	1.3574	0.104*	
H1C	0.220 (2)	0.4928 (10)	0.787 (2)	0.070 (6)*	

### Atomic displacement parameters (Å^2^)

**Table:**

	*U*^11^	*U*^22^	*U*^33^	*U*^12^	*U*^13^	*U*^23^
N1	0.0579 (11)	0.0482 (9)	0.0345 (9)	0.0048 (7)	0.0009 (7)	−0.0006 (7)
O1	0.0923 (11)	0.0558 (8)	0.0373 (8)	0.0027 (7)	−0.0070 (7)	−0.0063 (6)
N2	0.0593 (11)	0.0532 (9)	0.0348 (9)	0.0034 (8)	0.0009 (7)	−0.0044 (7)
N3	0.0710 (12)	0.0487 (10)	0.0317 (9)	0.0107 (8)	0.0045 (8)	0.0002 (7)
N4	0.0600 (12)	0.0826 (12)	0.0390 (9)	0.0162 (9)	0.0059 (8)	−0.0078 (8)
C7	0.0540 (13)	0.0530 (11)	0.0349 (11)	0.0074 (9)	0.0053 (9)	−0.0008 (9)
C8	0.0516 (12)	0.0470 (10)	0.0328 (10)	0.0056 (9)	0.0053 (9)	−0.0003 (8)
C11	0.0472 (12)	0.0533 (11)	0.0345 (11)	0.0033 (9)	0.0094 (9)	0.0002 (8)
C6	0.0535 (13)	0.0518 (11)	0.0412 (11)	0.0128 (9)	0.0107 (9)	0.0037 (9)
C18	0.0514 (12)	0.0450 (10)	0.0360 (11)	0.0013 (9)	0.0030 (9)	0.0020 (8)
C23	0.0496 (12)	0.0431 (10)	0.0374 (11)	−0.0043 (9)	0.0026 (9)	−0.0032 (8)
C9	0.0511 (12)	0.0519 (11)	0.0368 (11)	0.0043 (9)	0.0083 (9)	−0.0029 (9)
C12	0.0514 (13)	0.0478 (11)	0.0387 (10)	0.0043 (9)	0.0023 (9)	−0.0017 (8)
C19	0.0593 (14)	0.0597 (12)	0.0488 (13)	0.0046 (11)	0.0108 (10)	−0.0011 (9)
C22	0.0604 (14)	0.0598 (12)	0.0349 (11)	−0.0094 (11)	0.0021 (9)	0.0006 (9)
C20	0.0523 (14)	0.0586 (12)	0.0640 (15)	0.0066 (10)	0.0011 (11)	0.0047 (10)
C21	0.0582 (14)	0.0584 (12)	0.0479 (13)	−0.0035 (11)	−0.0058 (11)	0.0062 (10)
C10	0.0738 (15)	0.0611 (12)	0.0527 (13)	−0.0036 (11)	−0.0010 (11)	−0.0094 (10)
C5	0.0581 (14)	0.0749 (14)	0.0510 (13)	0.0100 (11)	0.0069 (11)	0.0136 (11)
C1	0.0972 (18)	0.0512 (12)	0.0512 (13)	0.0067 (12)	0.0052 (12)	−0.0013 (10)
C17	0.0594 (14)	0.0607 (13)	0.0559 (13)	−0.0024 (11)	0.0023 (11)	0.0036 (10)
C14	0.0823 (19)	0.0806 (17)	0.0775 (18)	0.0269 (14)	0.0038 (14)	−0.0240 (14)
C4	0.0871 (19)	0.0848 (17)	0.0628 (15)	0.0292 (14)	0.0128 (13)	0.0267 (14)
C16	0.0868 (19)	0.0544 (15)	0.0843 (18)	−0.0080 (12)	−0.0180 (15)	0.0113 (12)
C15	0.101 (2)	0.0552 (14)	0.097 (2)	0.0219 (15)	−0.0250 (17)	−0.0167 (15)
C2	0.143 (3)	0.0514 (14)	0.0723 (18)	0.0021 (14)	0.0209 (17)	−0.0010 (12)
C13	0.0708 (16)	0.0670 (13)	0.0543 (13)	0.0116 (11)	0.0137 (12)	−0.0078 (10)
C3	0.128 (2)	0.0603 (15)	0.0780 (18)	0.0322 (15)	0.0379 (17)	0.0195 (14)

### Geometric parameters (Å, °)

**Table:**

N1—C7	1.383 (2)	C22—H22A	0.9300
N1—N2	1.4031 (18)	C20—C21	1.377 (3)
N1—C6	1.417 (2)	C20—H20A	0.9300
O1—C7	1.2446 (19)	C21—H21A	0.9300
N2—C9	1.315 (2)	C10—H10A	0.9600
N3—C11	1.333 (2)	C10—H10B	0.9600
N3—C18	1.426 (2)	C10—H10C	0.9600
N3—H1C	0.96 (2)	C5—C4	1.376 (3)
N4—C23	1.376 (2)	C5—H5A	0.9300
N4—H4A	0.8600	C1—C2	1.387 (3)
N4—H4B	0.8600	C1—H1A	0.9300
C7—C8	1.446 (2)	C17—C16	1.381 (3)
C8—C11	1.397 (2)	C17—H17A	0.9300
C8—C9	1.429 (2)	C14—C15	1.368 (3)
C11—C12	1.482 (2)	C14—C13	1.377 (3)
C6—C1	1.381 (3)	C14—H14A	0.9300
C6—C5	1.387 (3)	C4—C3	1.369 (3)
C18—C19	1.378 (3)	C4—H4C	0.9300
C18—C23	1.396 (2)	C16—C15	1.371 (4)
C23—C22	1.402 (2)	C16—H16A	0.9300
C9—C10	1.493 (3)	C15—H15A	0.9300
C12—C17	1.383 (3)	C2—C3	1.368 (3)
C12—C13	1.390 (3)	C2—H2B	0.9300
C19—C20	1.380 (3)	C13—H13A	0.9300
C19—H19A	0.9300	C3—H3B	0.9300
C22—C21	1.361 (3)		
			
C7—N1—N2	111.75 (13)	C21—C20—H20A	120.3
C7—N1—C6	128.99 (15)	C19—C20—H20A	120.3
N2—N1—C6	119.23 (14)	C22—C21—C20	120.38 (18)
C9—N2—N1	106.48 (13)	C22—C21—H21A	119.8
C11—N3—C18	129.94 (16)	C20—C21—H21A	119.8
C11—N3—H1C	113.5 (11)	C9—C10—H10A	109.5
C18—N3—H1C	115.6 (11)	C9—C10—H10B	109.5
C23—N4—H4A	120.0	H10A—C10—H10B	109.5
C23—N4—H4B	120.0	C9—C10—H10C	109.5
H4A—N4—H4B	120.0	H10A—C10—H10C	109.5
O1—C7—N1	126.42 (16)	H10B—C10—H10C	109.5
O1—C7—C8	129.15 (16)	C4—C5—C6	119.6 (2)
N1—C7—C8	104.43 (14)	C4—C5—H5A	120.2
C11—C8—C9	132.03 (16)	C6—C5—H5A	120.2
C11—C8—C7	122.34 (15)	C6—C1—C2	119.4 (2)
C9—C8—C7	105.62 (14)	C6—C1—H1A	120.3
N3—C11—C8	118.40 (16)	C2—C1—H1A	120.3
N3—C11—C12	119.89 (15)	C16—C17—C12	119.7 (2)
C8—C11—C12	121.68 (15)	C16—C17—H17A	120.2
C1—C6—C5	119.43 (18)	C12—C17—H17A	120.2
C1—C6—N1	120.54 (17)	C15—C14—C13	120.7 (2)
C5—C6—N1	120.00 (17)	C15—C14—H14A	119.6
C19—C18—C23	120.54 (17)	C13—C14—H14A	119.6
C19—C18—N3	122.79 (17)	C3—C4—C5	121.4 (2)
C23—C18—N3	116.40 (16)	C3—C4—H4C	119.3
N4—C23—C18	120.83 (16)	C5—C4—H4C	119.3
N4—C23—C22	121.65 (17)	C15—C16—C17	121.0 (2)
C18—C23—C22	117.49 (18)	C15—C16—H16A	119.5
N2—C9—C8	111.47 (15)	C17—C16—H16A	119.5
N2—C9—C10	118.43 (16)	C14—C15—C16	119.3 (2)
C8—C9—C10	130.00 (17)	C14—C15—H15A	120.3
C17—C12—C13	119.14 (18)	C16—C15—H15A	120.3
C17—C12—C11	121.51 (18)	C3—C2—C1	121.3 (2)
C13—C12—C11	119.35 (18)	C3—C2—H2B	119.4
C18—C19—C20	120.60 (19)	C1—C2—H2B	119.4
C18—C19—H19A	119.7	C14—C13—C12	120.1 (2)
C20—C19—H19A	119.7	C14—C13—H13A	120.0
C21—C22—C23	121.51 (19)	C12—C13—H13A	120.0
C21—C22—H22A	119.2	C2—C3—C4	118.8 (2)
C23—C22—H22A	119.2	C2—C3—H3B	120.6
C21—C20—C19	119.4 (2)	C4—C3—H3B	120.6

### Hydrogen-bond geometry (Å, °)

**Table:**

*D*—H···*A*	*D*—H	H···*A*	*D*···*A*	*D*—H···*A*
N3—H1C···O1	0.96 (2)	1.93 (2)	2.733 (2)	139.8 (16)
N4—H4B···N2^i^	0.86	2.34	3.194 (2)	173.
N4—H4A···N2^ii^	0.86	2.50	3.209 (2)	140.

Symmetry codes: (i) *x*, *y*, *z*−1; (ii) −*x*+1, −*y*+1, −*z*+2.
